# Investigation of the radiation dose from cone‐beam CT for image‐guided radiotherapy: A comparison of methodologies

**DOI:** 10.1002/acm2.12239

**Published:** 2017-12-19

**Authors:** Jarryd G. Buckley, Dean Wilkinson, Alessandra Malaroda, Peter Metcalfe

**Affiliations:** ^1^ School of Physics Centre for Medical and Radiation Physics University of Wollongong Wollongong Australia; ^2^ Illawarra Cancer Care Centre Wollongong Hospital Wollongong Australia

**Keywords:** computed tomography dose index, cone beam computed tomography, gafchromic film, image guided radiotherapy, radiotherapy

## Abstract

Four methodologies were evaluated for quantifying kilovoltage cone‐beam computed tomography (CBCT) dose: the Cone‐Beam Dose Index (CBDI), IAEA Report 5 recommended methodology (IAEA), the AAPM Task Group 111 methodology (TG111), and the current dose metric; the Computed Tomography Dose Index (CTDI) on two commercial Varian cone‐beam CT imaging systems; the Clinac iX On‐Board Imager (OBI); and the TrueBeam X‐ray Imaging system (XI). The TG111 methodology measured the highest overall dose (21.199 ± 0.035 mGy OBI and 22.420 ± 0.002 XI for pelvis imaging) due to the full scatter of the TG111 phantom and was within 5% of CTDI measurements taken using a full scatter TG111 phantom and 30‐cm film strips. CBDI measured the second highest overall dose, within 10% of the TG111, with IAEA measuring the third highest dose. For head CBCT protocols, CBDI measured the highest dose, followed by IAEA. The CTDI method measured lowest across all scan modes highlighting its limitations for CBCT dosimetry. The XI imaging system delivered lower doses for head and thorax scan modes and similar doses to the OBI system for pelvis scan modes due to additional beam hardening filtration in the XI system. The TG111 method measured the highest dose in the center of a CBCT scan during image guidance procedures; however, CBDI provided a good approximation to TG111 with existing CTDI equipment and may be more applicable clinically.

## 1 INTRODUCTION

Modern radiotherapy has seen an increase in use of modulated dose delivery techniques such as intensity‐modulated radiation therapy (IMRT), volumetric modulated arc therapy (VMAT), and tomotherapy. With these new methods, the prescribed treatment dose can be delivered to the target with a high degree of conformity, while a steep dose gradient minimizes the dose to surrounding healthy tissue. It follows that variation in patient setup, anatomy, or movement during the course of treatment can lead to deviations in dose delivered to both the tumor and surrounding tissue from the original treatment plan. Hence, verification of patient setup at treatment is a fundamental step in ensuring precision in the delivery of radiotherapy.

Image guidance for patient positioning was originally performed using the megavoltage (MV) treatment beam and an electronic portal imaging device (EPID) or film placed behind the patient. However, at these MV energies, the inherent Compton scatter results in poor soft‐tissue contrast, limiting reference points within the body to higher Z tissue such as bone, or internal fiducial markers. To resolve this, many linear accelerators (LINACs) now have built‐in kilovoltage (kV) imaging systems that can produce images with improved soft‐tissue contrast to correct for internal organ motion and patient setup errors. Examples include the On‐Board Imager (OBI) and the TrueBeam X‐ray imaging system (XI) of Varian Medical Systems (Palo Alto, CA, USA). These devices consist of a kV X‐ray source and an amorphous silicon detector mounted to the LINAC gantry on extendable robotic arms orthogonal to the treatment beam. These devices can acquire 3D cone‐beam CT (CBCT) images of the patient in a single rotation of the gantry allowing registration with the radiotherapy planning CT to check for positional errors and make corrections as necessary with a high degree of accuracy.^1^, ^2^

Currently, imaging dose is often omitted from treatment plans since, being typically less than 1 Gy for an entire treatment schedule or 1–10 cGy for a single scan, it is two orders of magnitude smaller than the therapeutic doses.^1^, ^3^, ^4^, ^5^, ^6^, ^7^, ^8^, ^9^ However, during an imaging procedure, large portions of the body are irradiated, including radiosensitive structures such as lung, breast, thyroid, and reproductive organs. Bone structures also receive higher doses than other tissue at kV energies due to increased photoelectric absorption. Simulated doses in the femoral heads as high as 1.5–2.5 Gy have been reported due to daily pelvis CBCT imaging during a course of radiotherapy.^10^

Each clinic has its own protocols for frequency of CBCT imaging depending on tumor site and experience in day‐to‐day setup variations. While some radiotherapy clinics use daily CBCT imaging, often a typical CBCT schedule might be daily for the first week then once per week for the remainder of the treatment course. During a treatment of 30–40 fractions, the imaging dose has been shown to be significant, with reported effective doses of between 8 mSv and 46 mSv per CBCT scan leading to an increased risk of a patient developing a secondary primary malignancy.^1^, ^11^, ^12^ Therefore, a method for quantifying the imaging dose is necessary to evaluate any increased risk to the patient and aid in making informed decisions on the appropriate use of CBCT imaging during the course of treatment.

The traditional methods for quantifying fan‐bean CT dose, the Computed Tomography Dose Index (CTDI), underestimate CBCT dose due to an insufficient detector length to capture the full dose profile, and inadequate phantom length to achieve scatter equilibrium in the center of the detector.^13^ The underestimation worsens with increased beam width, which can be up to 40 cm for CBCT scans.^14^ Three alternative protocols have emerged in recent years which attempt to better quantify the imaging dose for wide beam scanning: The Cone‐Beam Dose Index (CBDI);^14^ The International Atomic Energy Agency (IAEA) Human Health Report No. 5;^15^ and the American Association of Physicists in Medicine (AAPM) Task Group 111 Report.^16^ While each protocol attempts to account for the limitations of CTDI in determining CBCT dose, their approach is somewhat different in terms of both equipment and measurement conditions. A comparison of all four methodologies for measuring CBCT dose forms the scope of this paper.

## 2 METHODS

### 2.A Phantom design and materials

**Figure 1:**
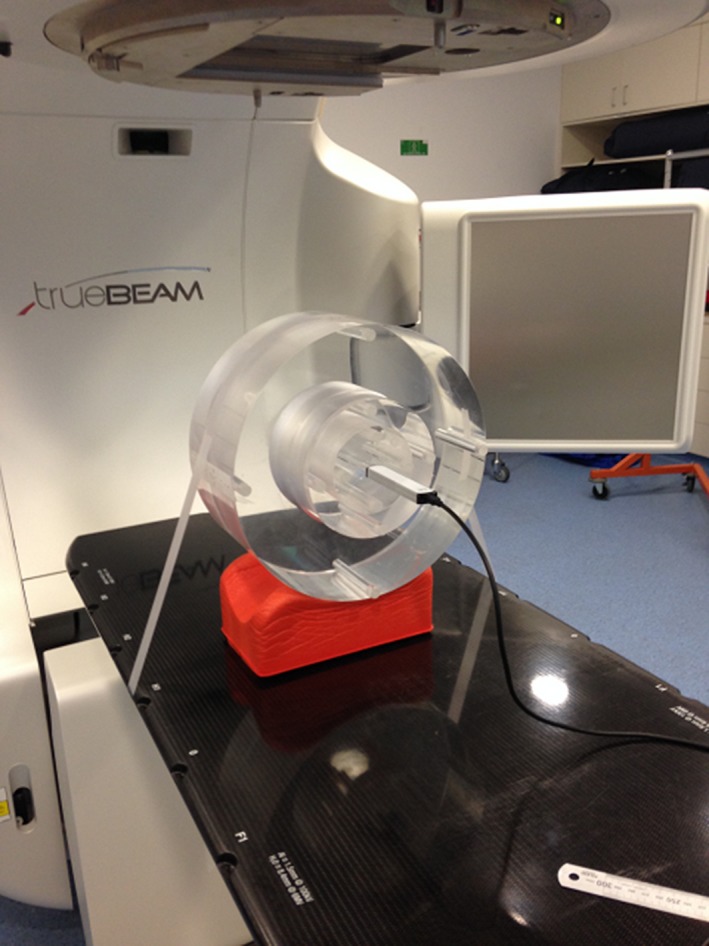
PMMA 32‐cm CTDI body phantom with the pencil chamber in the central position. For head protocols, the 16‐cm inner phantom was used.

A standard 32‐cm‐diameter poly methyl methacrylate (PMMA) CTDI body phantom and 16‐cm‐diameter head phantom were used for CTDI, CBDI, and IAEA measurements as shown in Fig. 1. Both phantoms have insert spaces for a 100‐mm pencil ionization chamber at the center and at the four peripheral locations. The phantom was placed on the couch with the center positioned at the isocenter using the room alignment lasers.

CTDI and CBDI measurements were taken using the UNFORS Xi detector system from RaySafe™. The system includes a base unit which connects to several detectors including a CT detector and HVL measurement tool for kV energies. The pencil ionization chamber for CT measurement has a sensitive length of 100 mm. The Xi device is a self‐contained detector, including the ionization chamber, electronics, and automatic temperature and pressure adjustments. The detector has a dose range of 10 μGy to 9999 Gy with an uncertainty of ±5%. Its energy dependence is <5% with an axial and radial uniformity of ±2% and ±3%, respectively.

**Figure 2:**
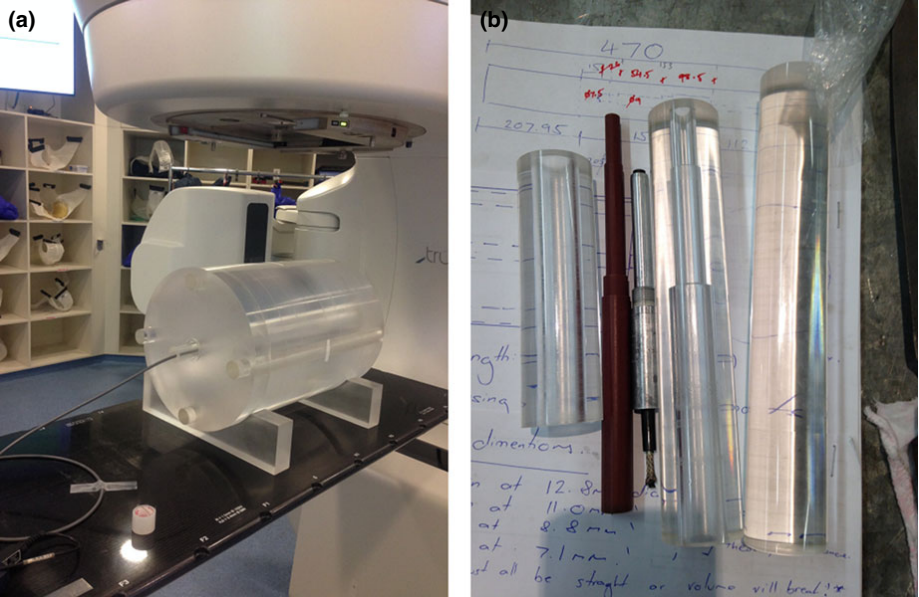
(a) The custom‐built TG111 phantom with a longitudinal length of 45 cm produces scatter equilibrium in the phantoms’ center. The cylindrical diameter of 32 cm is equivalent to the CTDI phantom and contains five plugs for weighted measurements. (b) Plug for housing the 0.6‐cc Farmer ionization chamber in the TG111 phantom. The plug contains three sections, one to run the cable out of the phantom, a middle section milled to conform to the chamber geometry, and a solid front section to fill the remaining air gap in the bore hole.

To fulfill the scatter requirements of the TG111 protocol, a new phantom was designed with a length of 45 cm [Fig. 2(a)]. The phantom was constructed from PMMA with the same 32 cm diameter as the CTDI phantom and five holes drilled into the phantom under the same center and peripheral configuration as the CTDI style measurements. During measurement, the ionization chamber was placed in one location, while the other four holes were filled with cylindrical PMMA plugs. The ionization chamber was housed in a customized plug, shown in Fig. 2(b), built to conform to the Farmer chamber geometry and eliminate air gaps around the sensitive volume.

The chamber used for the TG111 measurements was a 0.6‐cc NE 2571 Farmer ionization chamber. The sensitive air volume has a length of 24 mm and radius 3.2 mm. It is enclosed by a graphite thimble of thickness 0.065 g/cm^−2^. The chamber operates at a bias voltage of 300 V between the stem and chamber wall. The chamber was calibrated at the Australian Radiation Protection and Nuclear Safety Agency (ARPANSA) and is traceable to the Australian primary standard.

### 2.B OBI and XI CBCT imaging systems

Measurement of CBCT dose was performed on both a Varian 21iX on‐board imaging (OBI) and Varian TrueBeam X‐ray imaging (XI) systems. Each system can acquire 2D kV and 3D CBCT images as the source and detector are rotated around the patient. The clinical CBCT protocol settings used for the measurements in this study are given in Table 1.

**Table 1:** kV, mAs, and collimator settings for the clinical modes tested in the OBI and XI imaging systems

Protocol	kV	mAs	Superior–inferior (S–I) collimation (cm)	Axial collimation (cm)
Pelvis OBI	125	680	20.6	30.3
Pelvis XI	125	1056	21.4	28.1
Thorax OBI	110	262	20.6	30.3
Thorax XI	125	262	21.4	28.1
OBI high head	100	720	18.4	27.2
OBI std head	100	145	18.4	27.2
OBI low head	100	72	18.4	27.2
XI head	100	145	21.4	28.0

The beam width is modulated with independently adjustable X‐ and Y‐lead blade collimators. The field size at isocenter can be varied from 2.0 × 2.0 mm to 50.0 × 50.0 cm on both the XI and OBI systems. The X1 and Y1 collimators have a range of −25.0 to +3.5 cm and the X2, Y2 from −3.5 cm to +25 cm. On the XI system, a titanium beam hardening foil filter further hardens the X‐ray spectrum to reduce low‐energy photons. The axial plane is further modulated with an aluminum bow tie filter varying in thickness from 2 to 28 mm.

### 2.C Determining the CTDI and CBDI

Dose for a CBCT scan was measured with the pencil chamber placed sequentially in each position within the phantom. This dose value represents the average dose across the 100 mm length, and multiplying by the chamber length (*L*_c_) yields the dose‐length integral (DLI):

(1) $$\text{DLI}\left(\text{mGy}\cdot \text{mm}\right)=L_{c}\times D_{\mathrm{measured}}$$

where *D*_Measured_ represents the measured dose collected in scanning length *L*_c_ = 100 mm. To obtain the CTDI, the DLI is divided by the superior–inferior (S–I) collimation width (coll):

(2) $$\text{CTDI}\left(\text{mGy}\right)=\frac{1}{\text{coll}}\times \text{DLI}$$

The CBDI is calculated by dividing the DLI by the 100 mm sensitive length of the chamber *L*_c_:

(3) $$\text{CBDI}\left(\text{mGy}\right)=\frac{1}{L_{c}}\times \text{DLI}$$

Weighted CTDI_w_ and CBDI_w_ were then calculated from the CTDI_c_ measured in the center of the CTDI phantom and average of CTDI measurements in the peripheral positions CTDI_p_:

(4) $${\text{CTDI}}_{w}=\frac{1}{3}{\text{CTDI}}_{c}+\frac{2}{3}{\text{CTDI}}_{p}$$

The normalized ^n^CTDI_w_ and ^n^CBDI_w_ values, which represent CTDI_w_ per 100 mAs, were determined from the weighted CTDI_w_ values and corresponding mAs for the given scan:

(5) $${}^{n}{\text{CTDI}}_{w}=\frac{{\text{CTDI}}_{w}\times 100}{\text{mAs}}$$

The CBDI methodology proposed by Amer et al. stipulates additional scatter material be placed superior and inferior to the CTDI phantom to achieve scatter equilibrium in the center of the phantom. In this study, no additional scatter material was used for CBDI measurements. It should be noted that this will result in a reduction in measured dose, as reported by Amer et al.^14^

### 2.D IAEA methodology

The weighted IAEA_w_ dose was determined for the clinical protocols for pelvis, thorax, and head CBCT. As per the IAEA protocol for beam widths greater than 60 mm, a reference CTDI_ref_ is first determined with a S–I collimation of 2 cm. The CTDI_ref_ is an in‐phantom CTDI measurement with sufficiently narrow S–I collimation to facilitate the capture of the full dose profile by the 100‐mm pencil chamber within the CTDI phantom. The CTDI_ref_ is scaled by the ratio of CTDI, measured in free‐air, with S–I collimations of 2 cm and that used in the clinical protocol, to give IAEA_w_ as outlined in eq. (6):

(6) $${\text{IAEA}}_{w}={\text{CTDI}}_{\mathrm{ref}}\times \left(\frac{{\text{CTDI}}_{\mathrm{protocolwidth}}^{\mathrm{in}-\mathrm{air}}}{{\text{CTDI}}_{\mathrm{referencewidth}}^{\mathrm{in}-\mathrm{air}}}\right)$$

**Figure 3:**
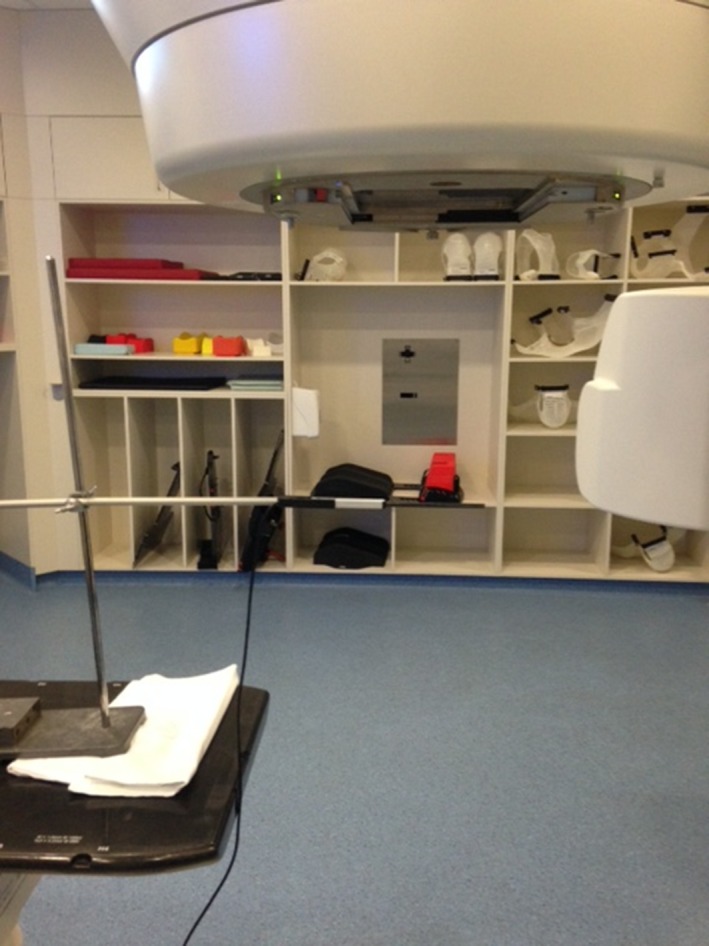
Experimental setup for the measurement of CTDI ^in‐air^. The 100‐mm chamber was stepped in 100‐mm increments to achieve the necessary integration length to capture the full dose profile. The chamber was held in place using a retort stand and rod. The chamber was extended from the couch a distance equal to half the total integration length to minimize scatter from the couch as specified by the IAEA.^15^

The free‐air measurements were taken with the pencil chamber suspended away from the couch as shown in Fig. 3. The kV, mAs, and axial collimation settings specified by the clinical protocol were applied for all measurements.

For free‐air measurements taken at the clinical CBCT S–I collimation, the IAEA specifies a minimum measurement length of the S–I collimation width + 40 mm, or 20 mm either side of the beam. For half‐fan protocols, the beam widths were 206 mm and 214 mm for the OBI and XI systems, respectively, corresponding to minimum measurement lengths of 246 mm and 254 mm. To achieve the required length, the pencil ionization chamber was stepped through the beam in three increments for a total measurement length of 300 mm. Preliminary measurements showed that even for a measurement length of 300 mm, a small fraction of the dose profile was being missed, likely due to scatter from the collimators. Hence, for clinical CBCT S–I collimation widths, the chamber was stepped through the beam in five increments for a total measurement length of 500 mm to capture the full dose profile.

The DLI for each chamber position was summed and divided by the S–I collimation to yield the protocol width CTDI^in‐air^:

(7) $${\text{CTDI}}_{\mathrm{protocol}\;\mathrm{width}}^{\mathrm{in}-\mathrm{air}}=\frac{\sum_{i=1}^{5}{\text{DLI}}_{i}}{\mathrm{coll}}$$

### 2.E AAPM TG111 methodology

The TG111 methodology for calculating CBCT dose is based on measuring dose in a phantom that provides close to full scattering conditions for broad cone‐beam imaging systems.^16^ As such, the custom‐built phantom described in Section A was used to determine a weighted TG111_w_ dose for the pelvis and thorax clinical CBCT protocols on both the OBI and XI systems. A separate head phantom was not constructed for our study, and hence, CBCT dose measurement using the TG111 approach was limited to the full 360° gantry rotation protocols. Dose was measured with a Farmer‐type cylindrical ionization chamber positioned centrally in the S–I axis of the phantom with the sensitive volume aligned to the isocenter. Similar to the CTDI measurements, TG111_w_ is determined by measuring dose at the center and four peripheral positions in the phantom.

The Farmer chamber used is traceable to the Australian primary dose standard through a series of air KERMA calibration factors (*N*_K_) for beams of a known HVL (Table 2). The HVL of the OBI and XI kV beams had previously been measured with the UNFORS detector. The accuracy of the UNFORS to measure HVL was verified on an orthovoltage unit for several beam qualities with well‐known HVL values. The charge collected (*q*) in the ionization chamber was corrected for ambient temperature and pressure and converted to dose using eq. (8):

(8) $$D_{w}\approx qN_{K}{\left(\frac{\overset{¯}{\mu }}{\rho }\right)}_{\mathrm{air}}^{\mathrm{material}}$$

where ${\left(\frac{\overset{¯}{\mu }}{\rho }\right)}_{\mathrm{air}}^{\mathrm{material}}$represents the ratio of spectrally averaged mass energy–absorption coefficients of the phantom material to air. For simplicity, and to be consistent with IAEA dose formalisms in diagnostic radiology,^17^ doses are quoted as dose to air and hence ${\left(\frac{\overset{¯}{\mu }}{\rho }\right)}_{\mathrm{air}}^{\mathrm{material}}=1$.

**Table 2:** HVL and corresponding *N*_K_ values for the 2571 Farmer chamber. *N*_K_ values were determined from a curve fit of the calibration certificate HVL to *N*_K_ data

Protocol	HVL (mm Al)	Correction factor *N*_K_ (mGy nC^−1^)
OBI pelvis	5.84	41.618
XI pelvis	8.43	41.591
OBI thorax	5.20	41.629
XI thorax	8.23	41.560

### 2.F Comparison of methodologies for increasing beam width and film measurements

To further investigate the limitations of the use of CTDIs for CBCT dosimetry, the four methodologies were evaluated for increasing S–I collimation widths. Measurements were taken at collimation widths of 2, 5, 10, 15, 20.6, 25, and 40 cm using the OBI clinical CBCT pelvis protocol. The S–I collimation setting for the OBI pelvis protocol of 20.6 cm was used in place of the standard 20‐cm measurement. The distance between successive measurements was increased for wider beams as scatter equilibrium was approached, and further increases in dose were minimal for the wider collimation widths. CTDI_w_, IAEA_w_, CBDI_w,_ and TG111_w_ values were determined at each collimation width.

The CBCT doses measured with the four different methodologies were also compared to a CTDI style integrated dose measurement CTDI_film_ using Gafchromic XR‐QA2 film. The film is sensitive in the energy range 20–200 kVp and a dose range of 0.1–20 cGy. The film consists of a 97‐μm polyester layer, 20‐μm adhesive layer, 25‐μm active layer, and a 97‐μm white polyester backing layer. Due to the strong energy dependence of the film for low energies, separate calibrations were performed for the OBI and XI imaging systems.^18^, ^19^, ^20^

The film was scanned prior to and 24 h postexposure on an Epson Expression 10000 XL flatbed scanner at 72 dpi resolution in 48‐bit color RGB mode. The scanner was operated in reflection mode with all corrections switched off. The images were analyzed in the red channel as this encompass the wavelength component associated with the most change in film color.^21^

The film was calibrated against air KERMA measured with a 0.6‐cc Farmer chamber with calibration traceable to a primary standard. To avoid changes in film response due to beam rotation, dose calibration for the film was performed in kV fluoroscopy mode with a stationary X‐ray tube.^19^, ^22^ The kVp, mAs, and collimator widths were set to be identical to the pelvis CBCT protocol for the calibration. Furthermore, the bow tie filter was inserted in place to ensure that the calibration was performed in the same beam quality as the CBCT beam.

The film was analyzed in ImageJ (National Institute of Health, Bethesda, MD, USA) and average pixel intensity across a 1 × 2 cm region of interest (ROI) for each piece used to determine net reflectance (net Δ*R*) using a method previously described by Tomic et al.^23^ The data were imported into MATLAB^®^ (MathWorks Inc., Natick, MA, USA) and a curve fit to the data. The applied fitting function was of the form $y=\frac{\mathrm{ax}}{b-x}$ where *x* and *y* represent net Δ*R* and air KERMA, respectively, with corresponding fitting parameters *a* and *b*. A fitting function of this form has the benefit of being monotonically increasing and returns a zero value for zero dose.

For CTDI_film_ measurements, 30 cm by 3 cm film strips were cut to measure the full length of the beam profile. A custom‐built PMMA cylindrical rod designed to fit in the holes of the TG111 phantom was created to house the film strips. The rod was cut into two hemispheres allowing the film to be placed in between the hemispheres before inserting the rod into the phantom. Individual strips were then exposed in each of the five positions within the TG111 phantom on OBI and XI systems. Two CBCT scans were acquired for each strip to deliver a higher dose to the film. The film processing and scanning procedure described earlier for the film calibration were maintained for the film strips. Line profiles were taken across the film strips and converted to air KERMA using the respective calibration curves. The converted air KERMA values were then halved to obtain the dose profile for a single CBCT scan. Due to the high sensitivity of XR‐QA2 film, two strips were irradiated and an average profile calculated. The center and peripheral DLIs were determined from the film profiles and divided by S–I collimation to yield the CTDI_film_ which was compared to TG111_w_ measurements on the OBI and XI systems.

## 3 RESULTS

### 3.A CTDI and CBDI measurements

Weighted CTDI_w_ and normalized ^n^CTDI_w_ dose for OBI and XI clinical protocols are shown in Table 3 with weighted CBDI_w_ and normalized ^n^CBDI_w_ dose shown in Table 4.

**Table 3:** Calculated CTDI_w_ and ^n^CTDI_w_ values for clinical CBCT modes on the OBI and XI systems. Uncertainties represent one standard deviation from repeated measurements

Clinical scan mode	CTDI_w_ (mGy)	^n^CTDI_w_ (mGy 100 mAs^−1^)
OBI pelvis	9.408 ± 0.065	1.384 ± 0.010
XI pelvis	9.646 ± 0.053	0.913 ± 0.005
OBI thorax	2.536 ± 0.006	0.968 ± 0.002
XI thorax	2.302 ± 0.010	0.879 ± 0.004
OBI high‐dose head	11.349 ± 0.058	1.576 ± 0.008
OBI std‐dose head	2.236 ± 0.021	1.542 ± 0.014
OBI low‐dose head	1.184 ± 0.019	1.644 ± 0.025
XI head	1.648 ± 0.010	1.137 ± 0.007

**Table 4:** Calculated CBDI_w_ and ^n^CBDI_w_ values for clinical CBCT modes on the OBI and XI systems. Uncertainties represent one standard deviation from repeated measurements

Clinical scan mode	CBDI_w_ (mGy)	^n^CBDI_w_ (mGy 100 mAs^−1^)
OBI pelvis	19.381 ± 0.135	2.850 ± 0.020
XI pelvis	20.642 ± 0.113	1.955 ± 0.011
OBI thorax	5.225 ± 0.013	1.994 ± 0.005
XI thorax	4.927 ± 0.021	1.880 ± 0.008
OBI high‐dose head	23.380 ± 0.106	3.247 ± 0.015
OBI std‐dose head	4.605 ± 0.038	3.176 ± 0.026
OBI low‐dose head	2.439 ± 0.033	3.388 ± 0.046
XI head	3.527 ± 0.020	2.432 ± 0.014

CTDI_w_ values were similar for OBI and XI pelvis protocols with XI measuring 0.24 mGy (3%) higher. The OBI thorax mode measured 0.23 (10%) mGy higher than the XI thorax mode. The varying mAs across the three OBI head modes were reflected in the respective doses, which varied by 10.17 mGy. OBI standard head mode measured 0.59 (36%) mGy higher than the XI head mode.

The normalized ^n^CTDI_w_ dose was higher for the OBI system across pelvis, thorax, and head protocols. In particular, the pelvis modes varied by 0.47 (43%) mGy. The difference is attributed to the higher mAs for pelvis scans on the XI system which is offset by its additional beam hardening titanium filter.

The trends described above for CTDI_w_ and ^n^CTDI_w_ dose also follow for CBDI as the CBDI simply upscales CTDI by dividing by chamber length rather than S–I collimation. Due to the upscaling, the CBDI values are more than double CTDI with an increase of 106% for OBI and 114% for XI protocols. These values would be higher still had additional scatter material been used for the CBDI.^14^

### 3.B IAEA measurements

The CTDI^in‐air^ dose and their ratios for the OBI and XI systems are presented in Table 5 and the weighted IAEA_w_ and normalized ^n^IAEA_w_ doses in Table 6. The OBI thorax mode could not be evaluated for the IAEA method as the UNFORS chamber would not trigger for reference beam width scans due to the low signal.

**Table 5:** Calculated CTDI_reference_ and CTDI_protocol_ in‐air values for clinical CBCT modes on the OBI and XI systems

Clinical scan mode	CTDI_reference width_ (mGy)	CTDI_protocol width_ (mGy)	Ratio^in‐air^
OBI pelvis	57.88	74.04	1.28
XI pelvis	67.10	69.13	1.03
XI thorax	16.42	16.94	1.03
OBI high‐dose head	38.73	47.33	1.22
OBI std‐dose head	7.89	9.49	1.20
OBI low‐dose head	4.08	5.02	1.23
XI head	5.11	5.29	1.04

**Table 6:** Calculated weighted IAEA_w_ and normalized ^n^IAEA_w_ values for clinical CBCT modes on the OBI and XI systems. Uncertainties represent one standard deviation from repeated measurements

Clinical scan mode	Weighted IAEA_w_ (mGy)	Normalized ^n^IAEA_w_ (mGy 100 mAs^−1^)
OBI pelvis	18.343 ± 0.001	2.698 ± 0.001
XI pelvis	14.771 ± 0.006	1.399 ± 0.001
XI thorax	4.441 ± 0.206	1.682 ± 0.078
OBI high‐dose head	15.316 ± 0.140	2.127 ± 0.019
OBI std‐dose head	3.747 ± 0.278	2.584 ± 0.192
OBI low‐dose head	1.726 ± 0.003	2.397 ± 0.004
XI head	2.810 ± 0.109	1.912 ± 0.075

The average in‐air ratio for the OBI system was 16% higher than for the XI system. The difference between OBI and XI systems is due to additional filtration in the XI system which removes low‐energy photons from the spectrum. Hence, the photon fluence in air is higher in the OBI system, and more energy is deposited within the pencil chamber.

The weighted pelvis IAEA_w_ was 3.57 (24%) mGy higher for the OBI system compared with XI. The OBI standard head mode was 0.937 (33%) higher than the XI head mode. The higher OBI values reflect the greater variation in dose measured between the reference beam width and protocol width resulting in a larger in‐air ratio for the OBI protocols when compared with the XI ones. These differences in ratios may partially be attributed to the additional low‐energy photon component that is filtered out by the titanium filter on the XI system. Normalized ^n^IAEA_w_ values varied by 1.30 (93%) mGy 100 mAs^−1^ and 0.04 (35%) mGy 100 mAs^−1^ across pelvis and head modes, respectively.

### 3.C TG111 measurements

The weighted TG111_w_ and normalized ^n^TG111_w_ doses for pelvis and thorax modes on the OBI and XI systems are given in Table 7.

**Table 7:** Calculated TG111_w_ and ^n^TG111_w_ values for pelvis and thorax clinical protocols on the OBI and XI imaging systems. Uncertainties represent one standard deviation from repeated measurements

Clinical scan mode	TG111_w_ (mGy)	^n^TG111_w_ (mGy 100 mAs^−1^)
OBI pelvis	21.199 ± 0.035	3.122 ± 0.005
XI pelvis	22.420 ± 0.002	2.123 ± 0.001
OBI thorax	6.963 ± 0.003	2.658 ± 0.001
XI thorax	5.540 ± 0.001	2.098 ± 0.001

The XI pelvis mode measured the highest dose, 1.22 (6%) mGy higher than the OBI pelvis protocol. The higher dose for XI can be attributed to a slightly larger transverse collimation width and a higher mAs. When normalized per 100 mAs, the OBI system delivered an additional 1.00 (47%) mGy compared with the normalized XI pelvis mode.

The OBI system measured 1.42 (26%) mGy higher than XI for the respective thorax modes and 0.56 (27%) mGy higher than XI for normalized ^n^TG111_w_. Both the OBI and XI systems deliver 262 mAs for thorax scanning; however, due to the titanium filter in the XI system, the fluence is higher in the OBI system with subsequently higher dose.

### 3.D Comparison of methodologies

**Figure 4:**
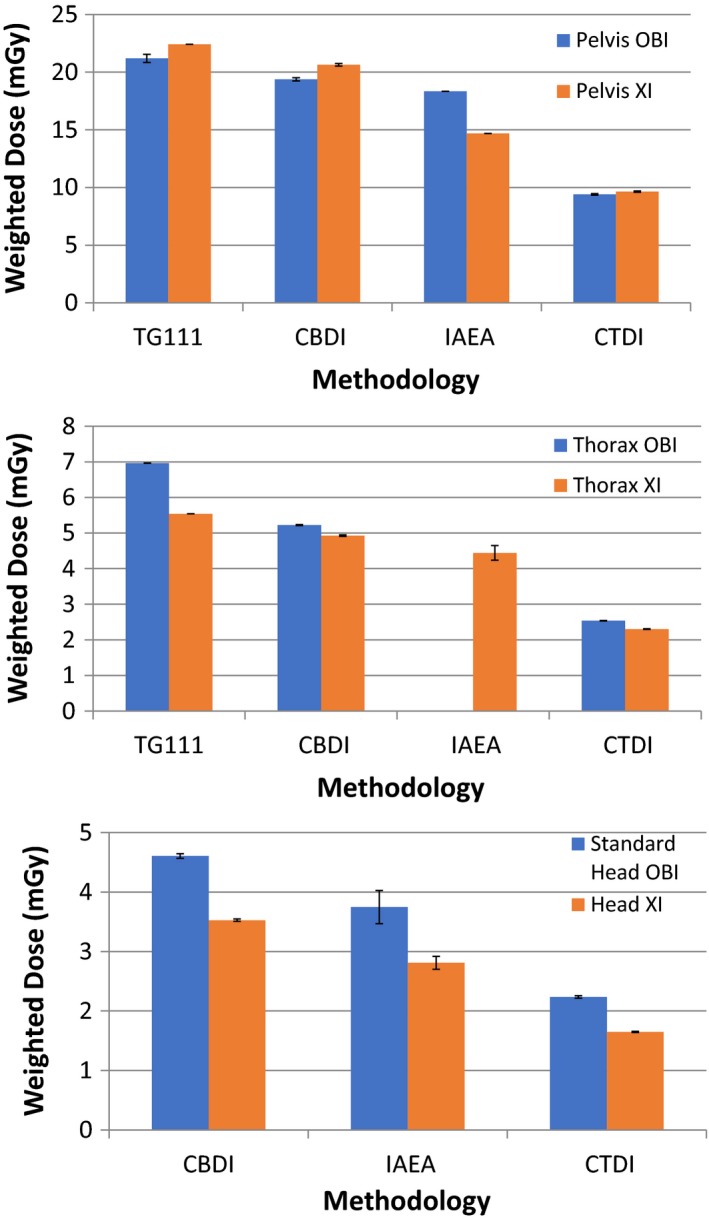
Comparison of TG111, CBDI, IAEA, and CTDI methodologies on the OBI and XI imaging systems for pelvis (a) and thorax (b) protocols. The IAEA was not evaluated for OBI thorax. Comparison of CBDI, IAEA, and CTDI methodologies for OBI standard head and XI head protocols is shown in (c). Error bars represent one standard deviation from repeated measurements.

Comparisons of TG111, CBDI, IAEA, and CTDI protocols for pelvis and thorax protocols are illustrated in Fig. 4(a) and 4(b). Comparison of CBDI, IAEA, and CTDI for head protocols are shown in Fig 4(c).

The TG111 methodology resulted in the highest recorded dose for the pelvis and thorax CBCT protocols. The CBDI methodology produced the second highest dose followed by the IAEA methodology, while the CTDI method yielded the lowest dose for each protocol. For pelvis modes, the CTDI_w_ measured 56% and 57% lower than TG111_w_ for OBI and XI, respectively, and similarly 64% and 58% lower for the thorax protocols.

For the head protocols and noting the absence of a TG111 measurement, the CBDI measured the highest dose, followed by IAEA and CTDI for the standard head OBI mode and XI head mode. The CTDI_w_ measured 106% and 114% lower than CBDI_w_ for OBI and XI head protocols, respectively.

### 3.E Comparison of methodologies for increasing beam width

**Figure 5:**
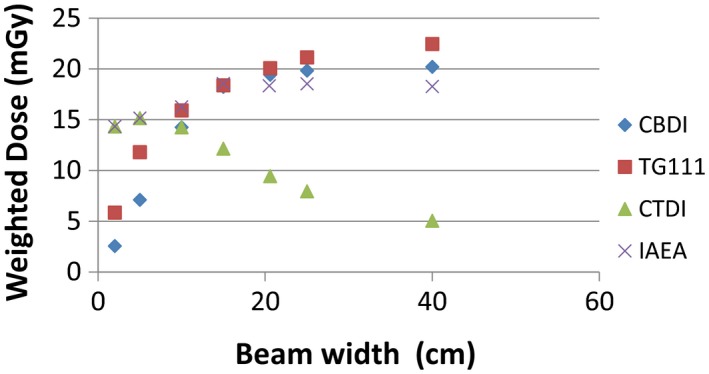
Weighted CTDI, CBDI, IAEA, and TG111 methodologies for increasing S–I collimation. The beam width was increased from 2 cm to 40 cm and was acquired on the OBI pelvis CBCT mode.

Weighted doses from CTDI, CBDI, IAEA, and TG111 protocols for S–I collimation widths ranging from 2 cm to 40 cm are presented in Fig. 5. The CTDI method yielded the highest dose for small beam widths with a peak value of 15.1 mGy at 5 cm. Further increases in beam width from 10 cm onward saw a decline in the CTDI such that a collimator width of 40 cm results in a CTDI dose value of 5.0 mGy. The IAEA method was equivalent to the CTDI for beam widths less than 10 cm. The IAEA reached a maximum of 18.5 mGy at 15 cm beam width and did not increase for beam widths beyond 15 cm. The CBDI method recorded the lowest dose for beam widths less than 10 cm as the measured dose is divided by the 10 cm chamber length, rather than the collimation width. At 10 cm beam width, the CBDI and CTDI both measured 14.2 mGy. The CBDI continued to increase asymptotically with a maximum value of 20.2 mGy at 40 cm collimation width. The TG111 method measured the second lowest dose for collimation widths less than 10 cm. At 10 cm beam width, the TG111 and IAEA methods agreed within 0.4 mGy. For wider collimation, the TG111 method increased asymptotically, but the increase continued for larger collimations compared to the CBDI due to the increased scatter provided by the 45 cm phantom. The TG111 reached a maximum value of 22.4 mGy at 40‐cm collimation width.

**Figure 6:**
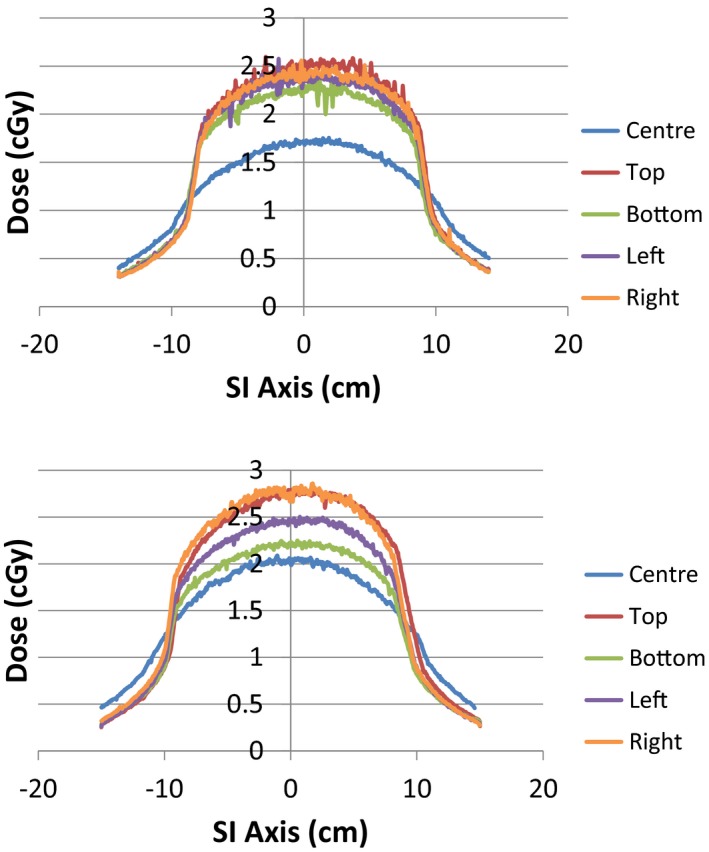
Film profiles in the center and peripheral positions within the TG111 phantom for OBI (a) and XI (b) systems.

Film profiles measured in the center and periphery of the TG111 phantom are shown in Fig. 6(a) for the OBI system and Fig. 6(b) for the XI system. The weighted CTDI_film_ values calculated from the film profiles for the OBI and XI systems are given in Table 8 along with the TG111_w_ values for comparison. The CTDI_film_ were within 3% and 5% of the TG111_w_ values for the OBI and XI systems, respectively. The film‐based doses were lower than the ionization chamber TG111_w_ doses in both cases.

**Table 8:** Calculated CTDI_film_ values and TG111_w_ values in mGy for OBI and XI systems. Uncertainties represent one standard deviation from repeated measurements

CBCT system	Weighted CTDI_film_ (mGy)	TG111_w_ (mGy)
Pelvis OBI	20.47 ± 0.71	21.20 ± 0.01
Pelvis XI	21.28 ± 0.44	22.42 ± 0.01

## 4 DISCUSSION

The TG111 methodology yielded the highest dose for pelvis and thorax CBCT protocols for both the OBI and XI systems. The higher TG111 doses are attributed to the longer TG111 phantom producing full scatter conditions. The approach to scatter equilibrium measured in the center of the phantom is observed in Fig. 5 for increasing collimation widths where TG111_w_ approaches a dose maximum at the largest S–I collimation width.

The CBDI method utilizing the existing CTDI phantom and pencil chamber demonstrated good agreement with the TG111 results for beam widths 10 cm and greater. However, as the collimation width of the beam increased beyond the 16 cm phantom length, a separation of the data points between the two methodologies is apparent (Fig. 5). The lower CBDI results at large collimator widths are attributed to the loss of scatter material beyond the edge of the phantom. In the original study by Amer et al.*,* when additional scatter material was placed at either end of the CTDI phantom, an increase of 31% and 8% at the center and periphery in CBDI was recorded, respectively.^14^

The IAEA approach captured the full dose profile in air which resulted in a higher value for CBCT dose compared with CTDI and, unlike CTDI, did not decrease for increasing beam width. The IAEA approach does underestimate the dose for CBCT scanning compared to TG111 and CBDI as it does not account for the contribution of scatter beyond the phantom for wide beams. The largest variation between IAEA with CBDI and TG111 was seen at the greatest collimation widths where scatter equilibrium was approached in the full‐length phantom. This is in contrast to the results from Hu and McLean, who reported in‐air correction factors of less than 1, which resulted in IAEA values more representative of CTDI.^7^ They concluded the IAEA method did not correct for efficiency losses due to the full beam width not being captured. A possible explanation could be the 300 mm in‐air integration length used by Hu for protocol width scans which did not capture the full dose profile. During our study, we found a 500 mm integration length was necessary to capture the full dose profile which was likely due to scattered photons from the collimators extending past the 300 mm integration length. Additionally, DLI calculated with a 300‐mm ionization chamber used in the Hu study averages dose across the full integration length, while for our approach, the dose integral was acquired from a summation of five dose integrals for each step of the pencil chamber. Lower doses at the ends of 300‐mm sensitive‐length chamber would reduce the average dose compared with a stepwise approach for which far ends of the detection length were weighted, in this case by $\frac{1}{5}$ of the dose integral as shown in eq. (7).

The CTDI values were significantly lower than the doses measured by the three alternative methodologies across all CBCT clinical protocols. Compared to TG111_w_, dose differences between 55% and 64% were observed for pelvis and thorax protocols across OBI and XI systems_._ The underestimation is due to an insufficient detector length to capture the full dose profile and a phantom without the required length to achieve full equilibrium scatter. The underestimation worsens with increasing beam width as the divisor in the CTDI calculation increases with minimal increase in the measured dose profile. This supports previous work from Boone who showed CTDI_100_ had an efficiency of 63% compared with CTDI with infinite detection and phantom length.^13^ Hu and McLean showed a 66% efficiency of a 100 mm integration length compared to a 300‐mm integration.^7^ Hu also demonstrated a dose difference of up to 36% between a 16‐cm phantom and 45‐cm phantom.

The slightly higher TG111_w_ measurements for the pelvis XI system compared to pelvis OBI were due to the higher mAs used for the XI system. The higher mAs in the XI system is offset by the beam hardening titanium foil filter which removes low‐energy photons from the X‐ray spectrum to reduce skin dose and improve signal‐to‐noise ratio. The normalized ^n^TG111_w_ values were higher for the OBI system. For thorax protocols, the OBI system delivered a higher dose than the XI system, where 262 mAs was used in both protocols. The higher ^n^TG111_w_ pelvis mode and TG111_w_ thorax mode doses for the OBI system can be attributed to the low‐energy photons present in the OBI beam.

The calculated CTDI_film_ dose showed good agreement (5% for XI and 3% for OBI) with the TG111_w_ values for both the OBI and XI systems. This result is consistent with the 3% agreement reported by Hu and McLean.^7^ The CTDI_film_ values were slightly lower than TG111 which is likely due to a small portion of the dose tail falling beyond the 30 cm sensitive length of the film, which was also reported by Hu, by comparing dose profiles measured along the full 45 cm phantom length with 30‐cm film strip measurements.

The doses measured in this study were in agreement with studies carried out using the same methodologies and imaging systems.^5^, ^7^, ^24^, ^25^ However, it has to be kept in mind that, depending on imaging system, software version and methodology, doses varied greatly from study to study, and this should be taken into account when interpreting the results.^7^, ^10^, ^16^, ^25^, ^26^, ^27^, ^28^, ^29^ Additionally, it must be stressed that doses presented in this work represent average air KERMA within a PMMA cylindrical phantom and should not be interpreted as patient dose. Any such conversion to patient dose would require information regarding the beam spectrum, organ site and patient size parameters.^30^

## 5 CONCLUSION

The methods evaluated in this work estimate the radiation output of two kV CBCT imaging systems as average dose to the center of a PMMA cylindrical phantom; they are therefore used as a tool to compare radiation exposures from different scanners and/or imaging protocols. This study investigated how the dose estimated by the AAPM TG111, the IAEA Report No. 5, and the Cone‐Beam Dose Index protocols, which try to account for higher S–I beam widths inherent with CBCT imaging, compares to the current standard for estimation of CT radiation output, the CTDI.

It has been shown that CTDI values systematically measured lower doses when compared to the three alternative methods; in particular, they underestimated doses when wider beam widths were considered. Amongst the protocols investigated, the TG111 method accounts for the full scatter profile using a longer cylindrical phantom than the other methods; it is therefore reasonable to consider the dose measured using the TG111 protocol as the best estimation of dose in the center of a PMMA phantom from a CBCT acquisition. This was supported by weighted average kV CBCT dose using CTDI_film_ profile measurements. The IAEA methodology agreed with the TG111 estimations in air, but it was not able to account for the full scatter profile when measured in a phantom. In the absence of a custom‐made full‐length phantom, the CBDI approach gives a comparable indication of CBCT dose to the TG111 methodology using equipment more commonly found in radiotherapy departments. Future work should also involve conversion of TG111 measurements to patient dose, taking into account patient‐specific imaging parameters and patient size.

As a secondary result of this study, it has been shown that, for the imaging protocols considered, the XI system consistently delivered lower dose than the OBI system due to its harder energy spectrum, in particular when values were normalized to 100 mAs.

## CONFLICT OF INTEREST

The authors declare no conflict of interest.

## References

[1] Kan MW , Leung LH , Wong W , Lam N . Radiation dose from cone beam computed tomography for image‐guided radiation therapy. Int J Radiat Oncol Biol Phys. 2008;70:272–279. 17980510 10.1016/j.ijrobp.2007.08.062

[2] Boda‐Heggemann J , Lohr F , Wenz F , Flentje M , Guckenberger M . kV cone‐beam CT‐based IGRT: a clinical review. Strahlenther Onkol. 2011;187:284–291. 21533757 10.1007/s00066-011-2236-4

[3] Ding GX , Coffey CW . Radiation dose from kilovoltage cone beam computed tomography in an image‐guided radiotherapy procedure. Int J Radiat Oncol Biol Phys. 2009;73:610–617. 19147025 10.1016/j.ijrobp.2008.10.006

[4] Giaddui T , Cui Y , Galvin J , Yu Y , Xiao Y . Comparative dose evaluations between XVI and OBI cone beam CT systems using Gafchromic XRQA2 film and nanoDot optical stimulated luminescence dosimeters. Med Phys. 2013;40:062102. 23718600 10.1118/1.4803466

[5] Islam MK , Purdie TG , Norrlinger BD , et al. Patient dose from kilovoltage cone beam computed tomography imaging in radiation therapy. Med Phys. 2006;33:1573–1582. 16872065 10.1118/1.2198169

[6] Kim S , Yoshizumi TT , Toncheva G , Yoo S , Yin F‐F . Comparison of radiation doses between cone beam CT and multi detector CT: TLD measurements. Radiat Prot Dosimetry. 2008;132:339–345. 19074786 10.1093/rpd/ncn305

[7] Hu N , McLean D . Measurement of radiotherapy CBCT dose in a phantom using different methods. Australas Phys Eng Sci Med. 2014;37:779–789. 25245234 10.1007/s13246-014-0301-x

[8] Kim S , Yoshizumi T , Toncheva G , Yoo S , Yin F‐F , Frush D . Estimation of computed tomography dose index in cone beam computed tomography: MOSFET measurements and Monte Carlo simulations. Health Phys. 2010;98:683–691. 20386198 10.1097/HP.0b013e3181cd3ec3

[9] Scandurra D , Lawford CE . A dosimetry technique for measuring kilovoltage cone‐beam CT dose on a linear accelerator using radiotherapy equipment. J Appl Clin Med Phys. 2014;15:80–92. 10.1120/jacmp.v15i4.4658 PMC5875512 25207398

[10] Spezi E , Downes P , Jarvis R , Radu E , Staffurth J . Patient‐specific three‐dimensional concomitant dose from cone beam computed tomography exposure in image‐guided radiotherapy. Int J Radiat Oncol Biol Phys. 2012;83:419–426. 22027261 10.1016/j.ijrobp.2011.06.1972

[11] Kim DW , Chung WK , Yoon M . Imaging doses and secondary cancer risk from kilovoltage cone‐beam CT in radiation therapy. Health Phys. 2013;104:499–503. 23532078 10.1097/HP.0b013e318285c685

[12] Halg RA , Besserer J , Schneider U . Systematic measurements of whole‐body imaging dose distributions in image‐guided radiation therapy. Med Phys. 2012;39:7650–7661. 23231313 10.1118/1.4758065

[13] Boone JM . The trouble with CTD100. Med Phys. 2007;34:1364–1371. 17500467 10.1118/1.2713240

[14] Amer A , Marchant T , Sykes J , Czajka J , Moore C . Imaging doses from the Elekta Synergy X‐ray cone beam CT system. Br J Radiol. 2007;80:476–482. 17684077 10.1259/bjr/80446730

[15] Agency IAE . Status of Computed Tomography Dosimetry for Wide Cone Beam Scanners. Vienna: International Atomic Energy Agency; 2011.

[16] Dixon R , Anderson J , Bakalyar D , et al. Comprehensive methodology for the evaluation of radiation dose in x‐ray computed tomography. *Report AAPM Task Group* 2010;111:20740–23846.

[17] Agency IAE . Dosimetry in Diagnostic Radiology: An International Code of Practice. Vienna: International Atomic Energy Agency; 2007.

[18] Tomic N , Quintero C , Whiting BR , et al. Characterization of calibration curves and energy dependence GafChromic XR‐QA2 model based radiochromic film dosimetry system. Med Phys. 2014;41:062105. 24877832 10.1118/1.4876295

[19] Giaddui T , Cui Y , Galvin J , Chen W , Yu Y , Xiao Y . Characteristics of Gafchromic XRQA2 films for kV image dose measurement. Med Phys. 2012;39:842–850. 22320794 10.1118/1.3675398

[20] Rampado O , Garelli E , Deagostini S , Ropolo R . Dose and energy dependence of response of Gafchromic XR‐QA film for kilovoltage x‐ray beams. Phys Med Biol. 2006;51:2871–2881. 16723772 10.1088/0031-9155/51/11/013

[21] Butson MJ , Cheung T , Yu PK . Absorption spectra of irradiated XRCT radiochromic film. Phys Med Biol. 2006;51:3099–3103. 16757865 10.1088/0031-9155/51/12/007

[22] Rampado O , Garelli E , Ropolo R . Computed tomography dose measurements with radiochromic films and a flatbed scanner. Med Phys. 2010;37:189–196. 20175481 10.1118/1.3271584

[23] Tomic N , Devic S , DeBlois F , Seuntjens J . Reference radiochromic film dosimetry in kilovoltage photon beams during CBCT image acquisition. Med Phys. 2010;37:1083–1092. 20384244 10.1118/1.3302140

[24] Hyer DE , Hintenlang DE . Estimation of organ doses from kilovoltage cone‐beam CT imaging used during radiotherapy patient position verification. Med Phys. 2010;37:4620–4626. 20964180 10.1118/1.3476459

[25] Ding GX , Munro P . Radiation exposure to patients from image guidance procedures and techniques to reduce the imaging dose. Radiother Oncol. 2013;108:91–98. 23830468 10.1016/j.radonc.2013.05.034

[26] Wen N , Guan H , Hammoud R , et al. Dose delivered from Varian's CBCT to patients receiving IMRT for prostate cancer. Phys Med Biol. 2007;52:2267–2276. 17404468 10.1088/0031-9155/52/8/015

[27] Alaei P , Spezi E . Imaging dose from cone beam computed tomography in radiation therapy. Phys Med. 2015;31:647–658. 26148865 10.1016/j.ejmp.2015.06.003

[28] Sykes J , Lindsay R , Iball G , Thwaites D . Dosimetry of CBCT: methods, doses and clinical consequences. J Phys: Conf Ser. 2013;44:012017.

[29] Palm Å , Nilsson E , Herrnsdorf L . Absorbed dose and dose rate using the Varian OBI 1.3 and 1.4 CBCT system. J Appl Clin Med Phys. 2010;11:229–240. 10.1120/jacmp.v11i1.3085 PMC5719770 20160695

[30] Boone J , Strauss K , Cody D , McCollough C , McNitt‐Gray M , Toth T . Size‐specific dose estimates (SSDE) in pediatric and adult body CT examinations. AAPM Report No. 204; 2011; http://www.aapm.org. Accessed 17/08/2017.

